# Scurvy Masquerading as Malignancy: A Diagnostic Challenge in a Child With Severe Selective Eating

**DOI:** 10.7759/cureus.111881

**Published:** 2026-07-01

**Authors:** Sara Babour, Soukaina Aithmadouch, Azzedine laaraj, Radi Abdelilah, Hassani Amale, Rachid Abilkassem

**Affiliations:** 1 Pediatric Rheumatology, Military Hospital Mohamed V, Rabat, MAR; 2 Pediatric Medicine, Military Hospital Mohamed V, Rabat, MAR; 3 Pediatric Critical Care, Military Hospital Mohamed V, Rabat, MAR

**Keywords:** diagnostic delay, gingival hypertrophy, limping, pediatric nutrition, scurvy, selective eating, vitamin c deficiency

## Abstract

Scurvy, caused by severe vitamin C deficiency, is increasingly being reported in modern developed countries, particularly among children with restrictive diets. The non-specific presentation often leads to extensive investigations and diagnostic delays.

We report the case of a 5.5-year-old boy born to consanguineous parents, with a history of language delay and severe selective eating, who presented with a two-week history of progressive limping and hemorrhagic gingival hypertrophy. Initial workup raised suspicion for hematologic malignancy, prompting extensive investigations, including bone marrow aspiration and maxillary biopsy, all of which were negative. A detailed dietary history revealed an extremely restricted diet consisting solely of pasteurized milk and biscuits for over three years. The vitamin C level was severely low at 3 µmol/L (normal range: 26-84 µmol/L), confirming the diagnosis of scurvy. Treatment with intravenous vitamin C (250 mg daily for one week) followed by oral supplementation resulted in dramatic improvement, with the patient regaining ambulation within 48 hours. Complete clinical and biochemical resolution occurred within three months.

This case highlights the critical importance of obtaining a detailed dietary history in children presenting with unexplained musculoskeletal symptoms, hemorrhagic manifestations, or failure to thrive. Scurvy remains a relevant diagnosis in modern pediatric practice, particularly among children with neurodevelopmental conditions and food selectivity. Early recognition can prevent unnecessary invasive procedures and expensive diagnostic workups.

## Introduction

Scurvy is a nutritional disorder resulting from prolonged and severe vitamin C (ascorbic acid) deficiency. Although historically associated with maritime exploration and previously considered a disease of antiquity, scurvy has re-emerged in contemporary pediatric practice across developed nations [1,2]. Recent literature documents an increasing incidence of pediatric scurvy, particularly among vulnerable populations, including children with autism spectrum disorder (ASD), developmental delays, selective eating behaviors, and socioeconomic barriers to adequate nutrition [3,4].

Humans cannot synthesize vitamin C endogenously due to the absence of the enzyme L-gulonolactone oxidase, making dietary intake essential [5]. Vitamin C serves critical functions in collagen synthesis, acting as a cofactor for prolyl and lysyl hydroxylases responsible for the post-translational modification of collagen. It additionally functions as a potent antioxidant, facilitates non-heme iron absorption, supports carnitine and catecholamine biosynthesis, and participates in immune regulation [5,6]. Clinical manifestations typically appear after one to three months of inadequate intake (less than 10 mg/day), once body stores fall below 300 mg [6].

The clinical spectrum of pediatric scurvy is protean, encompassing musculoskeletal complaints such as bone pain, refusal to ambulate, and pseudoparalysis; cutaneous manifestations including perifollicular hemorrhages, petechiae, and corkscrew hairs; oral findings such as gingival hypertrophy with hemorrhage; and systemic symptoms including fatigue, irritability, and failure to thrive [7,8]. This diversity of presentations, combined with the rarity of the condition in modern clinical practice, frequently results in diagnostic delays and extensive, costly investigations. Studies report that 80-86% of pediatric scurvy cases are initially misdiagnosed as other conditions, including malignancies, infections, or rheumatologic disorders [9,10].

Risk factors include neurodevelopmental disorders (particularly ASD), psychiatric conditions associated with food aversion, restrictive dietary patterns, malabsorptive disorders, and socioeconomic deprivation [11,12]. A particularly concerning observation is the association between severe selective eating - defined as consuming fewer than 20 different foods or avoiding entire food groups - and vitamin C deficiency, reported in up to 80% of children with ASD who develop scurvy [13].

This case report describes a child with severe selective eating presenting with atypical manifestations that mimicked hematologic malignancy, necessitating invasive diagnostic procedures before the correct diagnosis was established. We emphasize the diagnostic challenges posed by scurvy in the modern era and the critical importance of comprehensive nutritional assessment in the evaluation of children with unexplained systemic symptoms.

## Case presentation

A 5.5-year-old boy, the second of two children born to first-degree consanguineous parents residing in El Jadida, Morocco, presented to our pediatric emergency department with a two-week history of progressive limping affecting the left lower extremity. His birth history was unremarkable, with a birth weight of 3,250 g, length of 52 cm, and head circumference of 36 cm following an uncomplicated vaginal delivery at term. He had received exclusive breastfeeding for six months, followed by mixed feeding. Notably, the child had been followed by speech therapy since the age of four years for language delay. Growth parameters were consistently below -2 standard deviations (SD) for both weight and height throughout follow-up. Vaccination was complete according to Morocco's National Immunization Program. Parents denied any history of trauma, recent infections, or similar symptoms among family members or close contacts.

Timeline of presenting complaints

Two weeks before admission, the patient developed painless, progressive gingival hypertrophy localized to the maxillary region while remaining afebrile throughout this period. An outpatient consultation with a stomatologist led to further evaluation, including facial MRI, which revealed a 2.4 × 1.9 × 1.8 cm lesional process centered on the left maxillary bone. Biopsy of the maxillary lesion and excision of the gingival tissue were performed, demonstrating non-specific subacute inflammatory changes without evidence of malignancy. Complete blood count (CBC), erythrocyte sedimentation rate (ESR), and C-reactive protein (CRP) were within normal limits at that time.

One week before admission, progressive limping of the left lower extremity developed, accompanied by worsening gingival hypertrophy with spontaneous hemorrhage and increasing pain with hip mobilization. Plain radiographs of the hips and knees, along with hip ultrasonography, showed no abnormalities. The child was diagnosed with transient synovitis and prescribed nonsteroidal anti-inflammatory drugs (NSAIDs) without clinical improvement. By the time of admission, he had progressed to total functional impotence with complete inability to ambulate.

Physical examination

On examination, the patient appeared pale, asthenic, and uncomfortable but remained afebrile (temperature 37.2 °C). Anthropometric measurements revealed significant growth retardation, with a weight of 15 kg (weight-for-age *Z*-score: -2.3 SD) and height of 100 cm (height-for-age *Z*-score: -2.1 SD), according to the World Health Organization (WHO) Child Growth Standards [14]. Mid-upper arm circumference (MUAC) measured 12 cm, below the WHO threshold of 12.5 cm used to screen for acute malnutrition in children under five years; standardized MUAC cutoffs have not been validated for children older than five years, but this value remains consistent with significant nutritional compromise in this patient. No lymphadenopathy was appreciated. Musculoskeletal examination demonstrated complete inability to stand or walk independently, severe pain with passive mobilization of bilateral lower extremities, limited range of motion of the left hip with a heel-to-buttock distance exceeding 10 cm, bilateral knee flexion contractures (flessum deformity) preventing full extension, diffuse tenderness on palpation along the long bones, and diminished muscle strength of 4/5 in the lower extremities due to pain.

**Figure 1:**
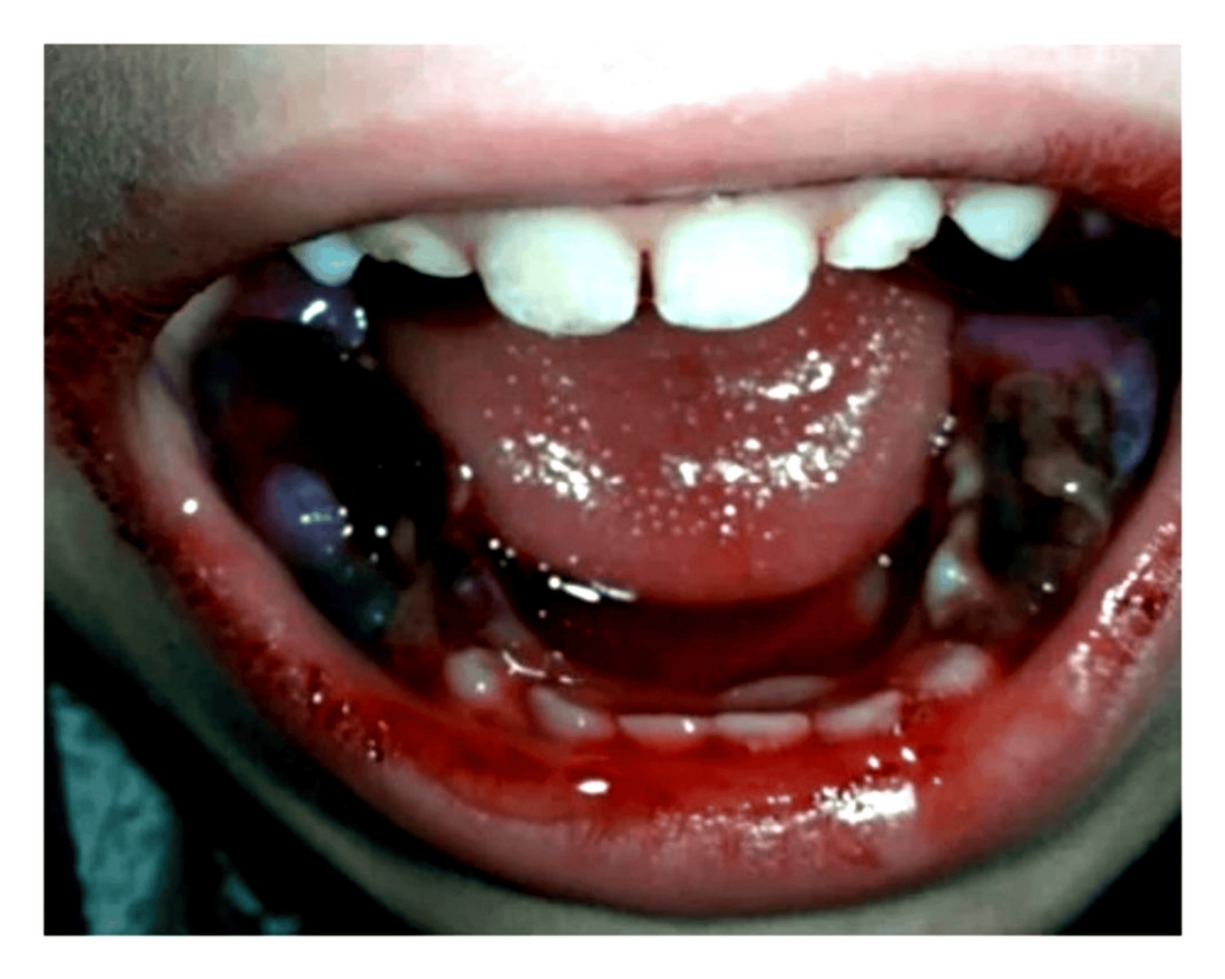
Hemorrhagic gingival hypertrophy at presentation (intraoral close-up view). Intraoral photograph demonstrating marked hemorrhagic gingival hypertrophy affecting both the maxillary and mandibular regions. The gingivae appear friable, edematous, and erythematous, with areas of spontaneous bleeding. The tissue has a spongy, boggy appearance characteristic of scurvy. Note the poor overall dentition and dental caries. This dramatic presentation initially raised concern for acute leukemic gingival infiltration, prompting an extensive hematologic workup, including bone marrow aspiration. The finding was completely resolved within one week of vitamin C supplementation, confirming its nutritional etiology and demonstrating the rapid reversibility characteristic of scurvy-related gingival changes.

**Figure 2:**
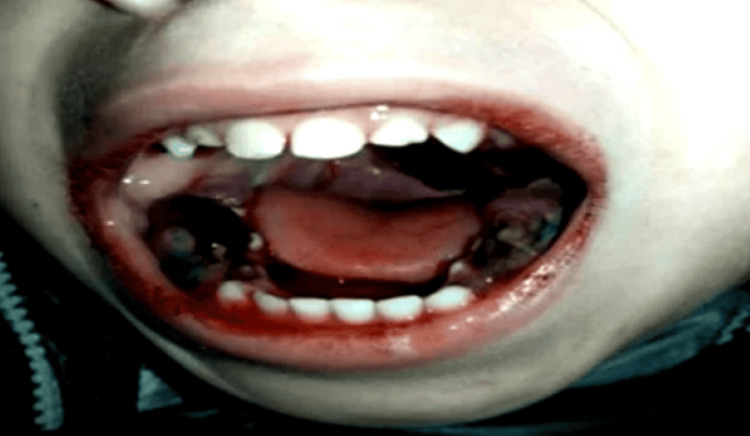
Gingival hypertrophy with active hemorrhage (full intraoral view). Full intraoral photograph showing extensive gingival hypertrophy with active hemorrhage affecting both dental arches. The gingivae demonstrate marked edema, erythema, and friability, with spontaneous bleeding evident on examination. The characteristic spongy texture and hemorrhagic appearance are characteristic of advanced scurvy. The presence of dental caries and poor oral hygiene likely contributed to secondary gingival inflammation superimposed on the vitamin C deficiency-related changes. This clinical presentation, combined with bone pain and petechiae, constituted a diagnostic triad that should have prompted earlier consideration of scurvy. Notably, the patient experienced no pain from the gingival lesions, which helped distinguish this from infectious gingivostomatitis.

Oral-facial examination revealed left-sided facial swelling in the maxillary region, marked gingival hypertrophy with active hemorrhage, and spongy, edematous gingivae with spontaneous bleeding tendency (Figures 1-2). Dentition was poor. Dermatologic examination disclosed a diffuse petechial rash predominantly involving the lower extremities and pallor of mucous membranes and conjunctivae. Corkscrew hairs were not initially identified but were later noted on close inspection. Cardiovascular, respiratory, and abdominal examinations were unremarkable, with no hepatosplenomegaly detected.

Diagnostic assessment

Given the constellation of findings - progressive limping, bone pain, gingival hemorrhage, petechiae, and a maxillary lesion - an extensive diagnostic workup was initiated to exclude malignancy, infection, and rheumatologic conditions (Table 1). Hematological investigations revealed a leukocyte count of 6,200/mm³, hemoglobin of 10.7 g/dL with a mean corpuscular volume of 71.5 fL and mean corpuscular hemoglobin of 24.5 pg, and a platelet count of 499,000/mm³. Peripheral blood smear showed hypochromia and microcytosis consistent with iron deficiency, without blast cells. Ferritin was low at 18 ng/mL (reference range: 32.9-336 ng/mL). Bone marrow aspiration demonstrated normal cellularity with normal maturation of all three cell lines and no evidence of malignant infiltration, effectively excluding leukemia**. **

**Table 1:** Estimated daily nutrient intake compared with age-appropriate RDA/DRI. Presents the estimated daily nutrient intake, calculated from the reported consumption of four to six 200-mL boxes of UHT-pasteurized milk and two to three packages of plain biscuits (~150 g/package) daily, using standard nutritional composition values for these food categories. This estimation, derived from dietary history rather than direct measurement, illustrates a paradoxical pattern of caloric and protein excess combined with near-total vitamin C deficiency, consistent with the calorie-dense, micronutrient-poor dietary pattern described in other pediatric scurvy cohorts. RDA, recommended dietary allowance; DRI, dietary reference intake

Nutrient	Estimated daily intake	RDA/DRI (age 4-8 years)	Recommendation (%)
Energy	~2,290 kcal	~1,200-1,400 kcal	~165-190% (excess)
Protein	~58 g	~19 g	~305% (excess)
Calcium	~1,200 mg	1,000 mg	~120% (adequate/excess)
Vitamin C	~5 mg	25 mg	~20% (severe deficiency)
Fruit/vegetable servings	0 servings	1-1.5 cups/day	0%

Inflammatory markers were only mildly elevated: ESR of 20 mm at the first hour and CRP of 15 mg/L. Protein electrophoresis revealed a total protein of 70 g/L and albumin of 33.9 g/L, consistent with a mild inflammatory pattern. The remainder of the biochemical profile, including renal function, hepatic enzymes, serum calcium, phosphorus, lipid panel, creatine phosphokinase, and lactate dehydrogenase (167 IU/L), was within normal limits. Vitamin B12 (602 pg/mL) and folate (4.10 ng/mL) were normal. Antinuclear antibodies, anti-double-stranded DNA, and complement levels were negative or normal. Viral serologies for HIV, hepatitis A/B/C, cytomegalovirus, Epstein-Barr virus, parvovirus B19, and herpes simplex virus were all negative.

**Figure 3:**
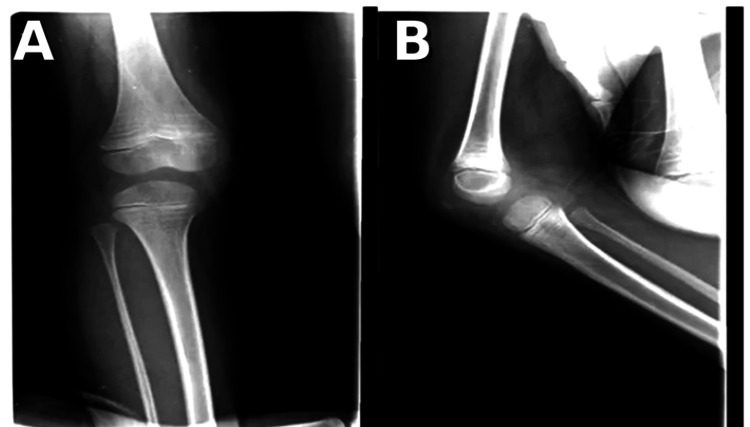
Lower extremity radiographs at presentation. Anteroposterior (Panel A) and lateral (Panel B) radiographs of the right knee were obtained at initial presentation. Retrospective review by an experienced pediatric radiologist, performed after confirmation of the diagnosis, identified subtle scorbutic changes that had initially been overlooked, including diffuse osteopenia, a ground-glass appearance of the metaphyses, pencil-thin cortices, and sclerotic metaphyseal lines consistent with Frankel's white line. These findings illustrate the context dependence of radiological interpretation in scurvy and underscore the importance of including the diagnosis in the differential before imaging review.

Radiological investigations, including plain radiographs of the hips, knees, and long bones, hip ultrasonography, whole-body bone scintigraphy with Tc-99m MDP, abdominal ultrasonography, and brain and spinal cord MRI, were unremarkable or non-contributory at initial interpretation (Figure 3). Histopathological examination of the maxillary bone and gingival biopsy specimens revealed a subacute non-specific inflammatory infiltrate without tumor cells or granulomatous features.

Dietary history and diagnostic breakthrough

Despite the extensive negative workup, the patient’s condition progressively worsened during the first week of hospitalization. He developed profound asthenia, becoming unable to sit upright or move voluntarily in bed. The appearance of a more extensive petechial rash prompted careful re-evaluation. On day 7 of hospitalization, a meticulous dietary history was elicited from the parents, representing the pivotal turning point in the diagnostic process.

The parents revealed that food diversification had been delayed until two years of age. From age two to 5.5 years, the child had consumed an exclusively restricted diet consisting of ultra-high temperature (UHT) pasteurized milk at four to six 200-mL boxes daily and plain biscuits at two to three packages daily, with absolute refusal of all other foods, including vegetables, fruits, meat, fish, eggs, and cheese. Multiple attempts at dietary diversification had consistently failed, often resulting in gagging, refusal to swallow, and extreme distress. Crucially, the parents confirmed no consumption of fresh fruits or vegetables for at least three years. As the UHT pasteurization process destroys most of the vitamin C content in milk, this dietary pattern left the child with virtually zero vitamin C intake.

Based on the clinical triad of severe dietary restriction, musculoskeletal symptoms with gingival hemorrhage, and petechiae, targeted testing was ordered. Serum ascorbic acid measured 3 µmol/L (reference range: 26-84 µmol/L), confirming severe vitamin C deficiency. Serum 25-hydroxyvitamin D was 18 nmol/L, indicating concurrent vitamin D deficiency. Retrospective review of the initial plain radiographs by an experienced pediatric radiologist, now performed with knowledge of the diagnosis, identified subtle findings consistent with scurvy that had been initially overlooked: diffuse osteopenia, ground-glass appearance of the metaphyses, pencil-thin cortices, and sclerotic metaphyseal lines (Frankel's white line) (Figure 3).

Treatment and clinical outcomes

Treatment was initiated immediately upon confirmation of the diagnosis. During the acute phase (week 1), the patient received intravenous ascorbic acid 250 mg once daily (approximately 17 mg/kg/day) for seven days, a single intramuscular dose of vitamin D3 300,000 IU for severe deficiency, oral iron supplementation for concurrent iron-deficiency anemia, daily oral multivitamin complex, and paracetamol as needed for pain management with discontinuation of NSAIDs.

Clinical improvement was rapid and dramatic. Within 48 hours of initiating treatment, pain levels improved significantly, the patient was able to sit up independently, and he began taking assisted steps. Gingival hemorrhage was substantially reduced. By one week post-treatment, the petechial rash had completely resolved, gingival hypertrophy had markedly decreased with cessation of hemorrhage, the patient was ambulating independently without pain, and his general energy and mood had dramatically improved.

At one month of follow-up, serum vitamin C had normalized to 52 µmol/L, the patient had gained 1.5 kg in weight, and his dietary repertoire had expanded to include 15 new foods, primarily fruits and vegetables. At three months, complete clinical resolution was confirmed: hemoglobin had normalized to 12.8 g/dL, vitamin D levels were 78 nmol/L, growth velocity had improved, and dietary variety had further expanded with dietitian support. Long-term management included continued multivitamin supplementation, regular follow-up with pediatric nutrition services, ongoing speech-language therapy addressing oral motor skills and feeding behaviors, and a scheduled autism spectrum disorder evaluation.

## Discussion

This case exemplifies the diagnostic challenges posed by pediatric scurvy in contemporary medical practice and underscores the critical importance of comprehensive nutritional assessment in children with unexplained multisystem symptoms. Our patient’s presentation - dominated by musculoskeletal complaints, gingival pathology, and petechiae - initially raised concern for hematologic malignancy, leading to an extensive and invasive diagnostic evaluation before the correct diagnosis was established through meticulous dietary history.

Epidemiology and risk factors

Although once considered a historical curiosity, scurvy has experienced a resurgence in modern developed nations [1,2]. Pediatric scurvy is increasingly recognized, particularly among high-risk populations, including children with neurodevelopmental disorders, socioeconomic disadvantage, and restrictive eating patterns [11,12]. Children with neurodevelopmental disorders, especially ASD, represent a particularly vulnerable group. Studies indicate that 30%-90% of children with ASD exhibit some degree of food selectivity, with a significant subset demonstrating severe restrictive eating patterns similar to our patient [13]. The *food neophobia *characteristic of many children with ASD - extreme reluctance to try new foods coupled with adherence to a narrow repertoire of preferred foods - significantly increases the risk of micronutrient deficiencies [4,13].

Our patient demonstrated several converging risk factors: severe dietary restriction lasting over three years, exclusive consumption of vitamin C-deficient foods (UHT-pasteurized milk and processed biscuits), language delay suggestive of a possible underlying neurodevelopmental condition, and parental consanguinity. Notably, the UHT pasteurization process destroys most vitamin C content in milk, leaving negligible amounts insufficient to meet daily requirements [8].

Pathophysiology

Vitamin C serves as an essential cofactor in multiple enzymatic reactions critical for human physiology [5]. Its primary role involves hydroxylation of proline and lysine residues in procollagen, a post-translational modification necessary for collagen triple helix stabilization [5]. Without adequate vitamin C, defective collagen synthesis results in impaired integrity of blood vessels, bones, skin, and connective tissues - the fundamental pathophysiologic basis of scurvy [7]. Additionally, vitamin C functions as a potent antioxidant, a cofactor in carnitine biosynthesis essential for fatty acid metabolism, a cofactor in catecholamine synthesis, an enhancer of non-heme iron absorption through reduction of ferric to ferrous iron, and a modulator of neutrophil function and immune response [5,6].

The body’s vitamin C pool approximates 1,500-2,000 mg in healthy individuals, with depletion occurring at a rate of 3%-4% daily [6]. Without dietary intake, clinical manifestations typically emerge after one to three months once plasma concentrations drop below 11 µmol/L [6]. Our patient’s extremely low level of 3 µmol/L indicated profound, long-standing depletion consistent with the chronicity of his dietary restriction.

Clinical manifestations and diagnosis

The clinical spectrum of pediatric scurvy is remarkably diverse, contributing to frequent misdiagnosis. Musculoskeletal symptoms constitute the most common presenting feature, occurring in 80%-96% of cases [7,9]. Our patient’s presenting complaint - limping progressing to complete inability to ambulate - represents a classic but often misinterpreted manifestation. The underlying pathophysiology involves subperiosteal hemorrhages causing intense bone pain, joint effusions, and hemarthroses [7]. Affected children adopt a *frog-leg* position with hips and knees flexed to minimize pain, a posture that may mimic septic arthritis or osteomyelitis [7,9]. Accordingly, the differential diagnosis must include septic arthritis, osteomyelitis, leukemia, rheumatologic disorders, and non-accidental trauma.

Gingival manifestations - including hypertrophy, sponginess, bleeding, and secondary infection - occur in 60%-75% of pediatric scurvy cases and were prominent in our patient [7,8]. Importantly, gingival changes occur only in dentate children, a useful clinical clue. Our patient’s dramatic gingival hypertrophy with hemorrhage initially suggested leukemia or infectious gingivostomatitis, illustrating the diagnostic confusion these findings can create.

Classic cutaneous findings include perifollicular hemorrhages, petechiae, and corkscrew hairs [7,8]. These result from capillary fragility secondary to defective collagen in vessel walls. Corkscrew hairs, while pathognomonic, were subtle in our case and identified only retrospectively on detailed examination; their absence should not exclude scurvy from consideration. Anemia, present in 42-80% of cases, is multifactorial, arising from decreased iron absorption, chronic blood loss from hemorrhage, and impaired red blood cell production [9,10]. Our patient’s iron-deficiency anemia and reactive thrombocytosis (platelets 499,000/mm³), reported in approximately 30% of scurvy cases, are consistent with published literature [9,10].

Plain radiography can reveal characteristic scorbutic changes, although early cases may appear normal. Reported findings include diffuse osteopenia, Frankel’s white line, the Trümmerfeld zone, Pelkan spur, and the Wimberger ring sign [7], as depicted in Figure 1. Retrospective review of our patient’s radiographs identified several of these features that had been initially overlooked, underscoring that radiological interpretation is heavily context-dependent. Advanced imaging, including MRI and bone scintigraphy, is not required for the diagnosis of scurvy and was performed in our case solely due to concern for malignancy, as illustrated in Figure 2.

Diagnostic delays and misdiagnosis

A striking aspect of our case was the prolonged diagnostic odyssey despite the presence of classical clinical features. Several factors contributed to this delay. First, rarity and unfamiliarity: most contemporary physicians have never encountered scurvy, and the diagnosis is rarely included in the initial differential [1,2]. Second, the protean manifestations of scurvy mimic numerous conditions, each requiring specific investigations [9,10]. Third, our patient’s nutritional appearance was misleading: his weight, though below 3-2 SD, did not suggest severe malnutrition. Studies show that 21%-36% of children with scurvy have normal or even elevated BMI, as they may consume calorie-dense but micronutrient-poor foods [15]. Fourth, pathognomonic signs were absent: corkscrew hairs are not universally present and may be subtle [8]. Fifth, the combination of bone pain, petechiae, anemia, and gingival hypertrophy appropriately raises concern for leukemia, delaying consideration of nutritional causes until invasive testing excludes malignancy [15,16]. Finally, and most critically, a detailed nutritional assessment - the single most important diagnostic step - was elicited only on day 7 of hospitalization [17].

Studies estimate the mean cost of diagnostic evaluation for pediatric scurvy patients presenting with musculoskeletal symptoms at approximately $14,000 per patient, ranging up to $80,000 in complex presentations [18]. This economic burden, combined with unnecessary exposure to invasive procedures and ionizing radiation, underscores the imperative for early recognition and the central role of dietary history in the clinical workup, as summarized in Table 1.

Treatment and prognosis

The management of scurvy is remarkably straightforward and uniformly successful. Treatment consists of vitamin C supplementation at 100-300 mg daily for children, with oral, intramuscular, or intravenous routes acceptable depending on severity [19]. Our patient received intravenous vitamin C 250 mg daily for one week due to the severity of his symptoms, followed by transition to oral therapy. Clinical improvement begins within 24-72 hours, with complete healing of gingival lesions and normalization of radiographic findings requiring several weeks to months [19]. Our patient’s timeline - ambulation restored within 48 hours and complete symptom resolution by one week - aligns with published literature [19].

Concurrent nutritional deficiencies, present in 22%-45% of scurvy cases, should be systematically identified and corrected [9,10,15]. Our patient required supplementation of vitamin D and iron, both commonly co-deficient in children with scurvy due to similarly restrictive diets, as detailed in Table 2.

**Table 2:** Comparison of patient findings with typical pediatric scurvy presentation. Compares the clinical, laboratory, and economic features of this case with those reported in the cited pediatric scurvy literature. The cited 80%-86% misdiagnosis rate and $14,000-$80,000 cost range represent population-level estimates from the referenced studies rather than figures specific to this patient; this patient's own diagnostic workup, including bone marrow aspiration, maxillary biopsy, whole-body bone scintigraphy, and brain/spinal MRI, totaled approximately $8,600 - within the lower portion of the reported range, but still substantial relative to the single low-cost serum ascorbic acid assay that ultimately confirmed the diagnosis. MRI, magnetic resonance imaging; BMI, body mass index

Feature	This patient	Typical pediatric scurvy (Literature)
Musculoskeletal symptoms	Present (limping, pseudoparalysis)	80%-96% of cases [7,9]
Gingival manifestations	Present (hypertrophy, hemorrhage)	60%-75% of cases [7,8]
Petechiae/cutaneous findings	Present; corkscrew hairs subtle, noted retrospectively	Classic but corkscrew hairs often absent/subtle [7,8]
Anemia	Present (iron-deficiency pattern)	42%-80% of cases [9,10]
Reactive thrombocytosis	Present (499,000/mm³)	~30% of cases [9,10]
BMI/nutritional appearance	Below -2 SD, not overtly malnourished-appearing	21%-36% normal/elevated BMI [14]
Concurrent micronutrient deficiency	Present (vitamin D, iron)	22-45% of cases [9,10,14]
Initial misdiagnosis	Suspected malignancy / transient synovitis	80%-86% initially misdiagnosed [9,10]
Approximate diagnostic workup cost	~$8,600	~$14,000-$80,000 [17]
Response to vitamin C treatment	Ambulation within 48 hours; resolution within 1 week	Improvement within 24-72 hours [18]

Long-term management and prevention

Beyond acute treatment, addressing the underlying cause - severe dietary restriction - is paramount to prevent recurrence. A multidisciplinary approach is essential, encompassing pediatric nutrition consultation for individualized dietary rehabilitation, speech-language pathology evaluation for oral motor and sensory assessment, developmental-behavioral pediatrics evaluation for underlying ASD or avoidant/restrictive food intake disorder (ARFID), and family education on balanced nutrition and food selectivity management [11,13,17].

Prevention remains the ultimate goal. Daily vitamin C intake recommendations are 15-25 mg for infants, 25-45 mg for children aged 1-8 years, and 45-75 mg for older children - amounts easily achieved through one to two daily servings of vitamin C-rich foods [6]. For children with persistent food selectivity, proactive multivitamin supplementation should be considered [13,19]. Primary care providers and pediatricians must incorporate routine dietary assessment into well-child visits, particularly for children with neurodevelopmental conditions or known food selectivity [11,17].

Comparison with published literature

Our case shares common features with published case series from multiple countries documenting the ongoing relevance of pediatric scurvy [1,2,9,10,15,16]. Like many reported cases, our patient had an underlying neurodevelopmental condition, restricted dietary variety limited to calorie-dense but vitamin C-poor foods, a significant diagnostic delay with invasive procedures, and a dramatic response to vitamin C supplementation [9,15], as summarized in Table 3. The association between scurvy and ASD deserves particular emphasis: multiple case series document selective eating leading to scurvy in children with ASD, and systematic reviews identify this population as the highest-risk group in high-income countries [4,11,13].

**Table 3:** Laboratory investigations summary. Comprehensive laboratory results were obtained at three time points: initial presentation (pre-diagnosis), day 7 of hospitalization (at diagnosis), and follow-up at one and three months post-treatment. Reference ranges are provided for each parameter. Directional arrows indicate deviation from normal: ↓ = mildly decreased; ↓↓ = severely decreased; ↑ = mildly increased. The serum ascorbic acid level of 3 µmol/L (reference: 26-84 µmol/L), obtained on day 7 following elicitation of a detailed dietary history, was critically low and confirmed the diagnosis of scurvy. Additional findings included microcytic hypochromic anemia consistent with concurrent iron deficiency (hemoglobin 10.7 g/dL, ferritin 18 ng/mL), reactive thrombocytosis (platelets 499,000/mm³), and severe vitamin D deficiency (25-hydroxy (25-OH) vitamin D 18 nmol/L). Inflammatory markers were only mildly elevated (CRP 15 mg/L, ESR 20 mm/h), and bone marrow aspiration was normal, excluding hematologic malignancy. All deficiencies normalized following targeted supplementation, with hemoglobin reaching 12.8 g/dL and vitamin C normalizing to 52 µmol/L at one month. MCV, mean corpuscular volume; MCH, mean corpuscular hemoglobin; ESR, erythrocyte sedimentation rate; CRP, C-reactive protein

Parameter	Value at presentation	Reference range	Interpretation
Hematology			
Hemoglobin	10.7 g/dL	11.5-15.5 g/dL	↓ Mild anemia
MCV	71.5 fL	77-95 fL	↓ Microcytic
MCH	24.5 pg	25-33 pg	↓ Hypochromic
Leukocytes	6,200/mm³	5,000-15,000/mm³	Normal
Platelets	499,000/mm³	150,000-450,000/mm³	↑ Thrombocytosis
Ferritin	18 ng/mL	32.9-336 ng/mL	↓ Iron deficiency
Inflammatory markers			
ESR	20 mm/h	<15 mm/h	↑ Mild elevation
CRP	15 mg/L	<10 mg/L	↑ Mild elevation
Biochemistry			
Albumin	33.9 g/L	35-50 g/L	↓ Mild hypoalbuminemia
Calcium	102 mg/L	95-105 mg/L	Normal
Phosphorus	55 mg/L	30-60 mg/L	Normal
Alkaline phosphatase	305 U/L	150-420 U/L	Normal
Micronutrients			
Vitamin C (ascorbic acid)	3 µmol/L	26-84 µmol/L	↓↓ Severe deficiency
25-OH, Vitamin D	18 nmol/L	50-250 nmol/L	↓ Deficiency
Vitamin B12	602 pg/mL	200-900 pg/mL	Normal
Folate	4.10 ng/mL	2.7-17.0 ng/mL	Normal

The *beige diet* - predominantly milk, bread, pasta, and crackers - is commonly described in published series and mirrors our patient’s diet [11,13]. Toscano et al. recently reported five Italian children with scurvy, all with neurodevelopmental disorders, including one with life-threatening pulmonary hypertension, highlighting the potential for severe complications [10]. Miraj et al. found that 86% of 18 Indonesian cases were initially misdiagnosed, with osteomyelitis being the most common erroneous diagnosis [9] - a pattern identical to our case’s initial differential.

Limitations

This case report has several limitations. As a single-patient study, generalizability is inherently limited. No formal ASD evaluation had been completed at the time of scurvy diagnosis, though language delay and extreme food selectivity raise clinical suspicion for an underlying neurodevelopmental condition. Long-term follow-up beyond three months was not available at the time of manuscript preparation. Formal feeding evaluation, including videofluoroscopic swallow study, was not performed to exclude oral motor dysfunction contributing to dietary restriction. Finally, systematic measurement of micronutrients beyond iron and vitamin D was not undertaken, and additional deficiencies may have been present but undetected.

## Conclusions

Scurvy, though historically associated with maritime exploration and malnutrition, remains clinically relevant in modern pediatric practice, particularly among children with neurodevelopmental conditions, developmental delays, language disorders, or psychiatric conditions that predispose to severe food selectivity. This case underscores the critical importance of maintaining a high index of suspicion for nutritional deficiencies when evaluating children with unexplained musculoskeletal pain, hemorrhagic manifestations, gingival changes, or multisystem symptoms of unclear etiology. A comprehensive dietary history - including specific inquiry about fruit and vegetable intake - should be considered standard practice in such presentations, as this simple, cost-free step could have prevented the extensive and invasive diagnostic workup our patient underwent. Early recognition not only spares children from unnecessary procedures and radiation exposure but also enables prompt initiation of curative treatment, with most patients achieving ambulation within 24 to 72 hours and complete clinical resolution within weeks of vitamin C supplementation.

Beyond individual case management, this case highlights a broader public health concern. In an era of abundant food availability in developed nations, the re-emergence of scurvy serves as a sobering reminder that nutritional deficiencies persist among vulnerable populations. Long-term success requires a multidisciplinary approach addressing the underlying dietary restriction through nutritional counseling, behavioral feeding therapy, and management of co-existing conditions to prevent recurrence. Heightened awareness among healthcare providers, systematic nutritional screening in high-risk children, and early intervention are essential to prevent significant morbidity from this eminently treatable condition.
